# Whole-Exome Sequencing for Identification of Genetic Variants Involved in Vitamin D Metabolic Pathways in Families With Vitamin D Deficiency in Saudi Arabia

**DOI:** 10.3389/fgene.2021.677780

**Published:** 2021-06-08

**Authors:** Shatha Alharazy, Muhammad Imran Naseer, Eman Alissa, Margaret Denise Robertson, Susan Lanham-New, Adeel G. Chaudhary

**Affiliations:** ^1^Department of Physiology, Faculty of Medicine, King Abdulaziz University, Jeddah, Saudi Arabia; ^2^Center of Excellence in Genomic Medicine Research, King Abdulaziz University, Jeddah, Saudi Arabia; ^3^Department of Medical Laboratory Technology, Faculty of Applied Medical Sciences, King Abdulaziz University, Jeddah, Saudi Arabia; ^4^Department of Clinical Biochemistry, Faculty of Medicine, King Abdulaziz University, Jeddah, Saudi Arabia; ^5^Department of Nutritional Sciences, Faculty of Health and Medical Sciences, University of Surrey, Guildford, United Kingdom; ^6^Centre for Innovation in Personalized Medicine, King Abdulaziz University, Jeddah, Saudi Arabia

**Keywords:** Saudi Arabia, family study, variants, polymorphisms, vitamin D, whole-exome sequencing, vitamin D metabolism, vitamin D deficiency

## Abstract

**Background:**

Numerous research studies have found an association between vitamin D (vitD) status and single-nucleotide polymorphisms (SNPs) in genes involved in vitD metabolism. It is notable that the influence of these SNPs on 25-hydroxyvitamin D [25(OH)D] levels might vary in different populations. In this study, we aimed to explore for genetic variants in genes related to vitD metabolism in families with vitD deficiency in Saudi Arabia using whole-exome sequencing (WES).

**Methods:**

This family-based WES study was conducted for 21 families with vitD deficiency (*n* = 39) in Saudi Arabia. WES was performed for DNA samples, then resulting WES data was filtered and a number of variants were prioritized and validated by Sanger DNA sequencing.

**Results:**

Several missense variants in vitD-related genes were detected in families. We determined two variants in low-density lipoprotein 2 gene (LRP2) with one variant (rs2075252) observed in six individuals, while the other LRP2 variant (rs4667591) was detected in 13 subjects. Single variants in 7-dehydrocholesterol reductase (DHCR7) (rs143587828) and melanocortin-1 receptor (MC1R) (rs1805005) genes were observed in two subjects from two different families. Other variants in group-specific component (GC), cubilin (CUBN), and calcium-sensing receptor (CASR) gene were found in index cases and controls. Polymorphisms in GC (rs9016) and CASR (rs1801726) were found in the majority of family cases (94 and 88%), respectively.

**Conclusion:**

In vitD-deficient families in Saudi Arabia, we were able to detect a number of missense exonic variants including variants in GC (rs9016), CUBN (rs1801222), CASR (rs1801726), and LRP2 (rs4667591). However, the existence of these variants was not different between affected family members and non-affected controls. Additionally, we were able to find a mutation in DHCR7 (rs143587828) and a polymorphism in LRP2 (rs2075252), which may affect vitD levels and influence vitD status. Further studies are now required to confirm the association of these variants with vitD deficiency.

## Introduction

Vitamin D (vitD) plays an important role in maintaining skeletal calcium (Ca) homeostasis by stimulating intestinal absorption of Ca and phosphate (PO4), stimulating bone resorption and inducing Ca reabsorption by the kidney, thus sustaining the level of calcium and phosphate necessary for bone formation and supporting appropriate functioning of parathyroid hormone (PTH) to maintain Ca levels in serum (Holick, 2007; Holick et al., 2011).

Clinically, serum 25-hydroxyvitamin D [25(OH)D] has been identified as the most effective predictor of vitD status, to date. Levels of 25(OH)D in serum are influenced by the vitD produced dermally and consumed orally, through diet or supplementation (Hollis, 1996; Del Valle et al., 2011). In addition, there are physiological, pathological, and lifestyle factors affecting 25(OH)D levels such as aging, obesity, liver and kidney diseases, and inadequate exposure to sunlight (Holick, 2004, 2007; Tsiaras and Weinstock, 2011; Hyppönen and Boucher, 2018). Among other significant factors influencing 25(OH)D levels are the genetic factors with the heritability of circulating 25(OH)D levels predicted to be between 23 and 80% (Bahrami et al., 2018), primarily as single-nucleotide polymorphisms (SNPs) in genes involved in the vitD metabolic pathway (Ahn et al., 2010; McGrath et al., 2010; Wang et al., 2010; Jolliffe et al., 2016).

Vitamin D metabolism undergoes numerous pathways that are genetically regulated. VitD is produced mainly in the skin epidermis under the effect of ultraviolet-B radiation which activates 7-dehydrocholesterol (7-DHC) in the skin to form pre-vitD (Datta et al., 2017). 7-DHC is converted to cholesterol by the enzyme 7-dehydrocholesterol reductase (DHCR7) and the presence of the reduced coenzyme nicotinamide adenine dinucleotide phosphate (NAD-P) which is activated by nicotinamide adenine dinucleotide synthetase 1 (NADSYN1) (Ahn et al., 2010). VitD (from all sources) is hydroxylated in the liver mainly by cytochrome p450 enzyme (CYP2R1/25-hydroxylase) to form 25(OH)D, with further hydroxylation in the kidney distal tubule by cytochrome p450 enzyme (CYP27B1/1α-hydroxylase) producing 1,25-dihydroxyvitamin D [1,25(OH)_2_D], the active form of vitD (Eyles et al., 2005). Both 25(OH)D and l,25(OH)_2_D are eliminated under the effect of another cytochrome P450 enzyme (CYP24A1/24-hydroxylase), transforming them into inactive waste calcitroic acid products [24,25(OH)_2_D and 1,24,25(OH)_3_D] (McGrath et al., 2010). Metabolites of vitD including 25(OH)D and 1,25(OH)_2_D are primarily transported by vitamin D-binding protein (VDBP) encoded by group-specific components (GCs) (Daiger et al., 1975; Speeckaert et al., 2006). Cubilin (intrinsic factor-cobalamin receptor) and Magalin (low-density lipoprotein-related receptor), which are encoded by the cubilin gene (CUBN) and low-density lipoprotein 2 gene (LRP2), respectively, assist renal cells in the uptake of the 25(OH)D VDBP complex (McGrath et al., 2010; Kaseda et al., 2011; Kohlmeier, 2015). The active form of vitD, 1,25(OH)_2_D, initiates its genomic action by binding to the vitamin D receptor (VDR) (Pike and Meyer, 2012). In addition to these genes that can be directly implicated in vitD metabolism, additional genes such as the calcium-sensing receptor (CASR) that contributes in vitD metabolism by regulating PTH and Ca levels (Brown, 2013) may also be clinically important in determining vitD status.

Vitamin D deficiency is highly prevalent in Saudi Arabia. Although several studies have already reported an association between vitD status and SNPs in genes involved in vitD metabolism (McGrath et al., 2010; Jolliffe et al., 2016), the influence of these SNPs on 25(OH)D levels might vary in different populations. For example, an SNP in DHCR7 (rs12800438) was related to vitD deficiency in African Americans but not in European Americans (Batai et al., 2014), and another SNP in DHCR7 (rs12785878) was linked to vitD deficiency in Chinese cohorts from Kazak ethnicity but not in Uyghurs (Xu et al., 2015).

The relationship between inherited variants in vitD-related genes and vitD deficiency has not been adequately addressed in Saudi Arabia. Whole-exome sequencing (WES) analysis is designated as state-of-art, sequencing large amounts of DNA with high throughput, providing fast and broad data about known or novel mutations in candidate genes in family members with a specific disease or trait. Therefore, we aimed to investigate the presence of genetic variants in genes related to vitD metabolism among families with vitD deficiency in Saudi Arabia using WES.

## Materials and Methods

### Study Design and Recruitment

Members from families with a history of vitD deficiency were recruited for this study from a single tertiary center [King Abdulaziz University Hospital (KAUH), Jeddah, Saudi Arabia] and seven primary health care centers (PHCCs) distributed in Jeddah (a PHCC from each of the seven sectors of Jeddah area). The study was undertaken at the Center of Innovation in Personalized Medicine (CIPM), King Fahd Medical Research Center (KFMRC), in Jeddah.

The favorable ethical opinion of this study was provided by the Research Ethics Committee in Unit of Biomedical Ethics, Center of Excellence in Genomic Medicine Research (CEGMR), King Abdulaziz University (KAU) (ref no. 05-CEGMR-Bioeth-2018). All participants provided written informed consent, with both parental consent and child assent obtained for participants under 16 years of age.

**FIGURE 1:**
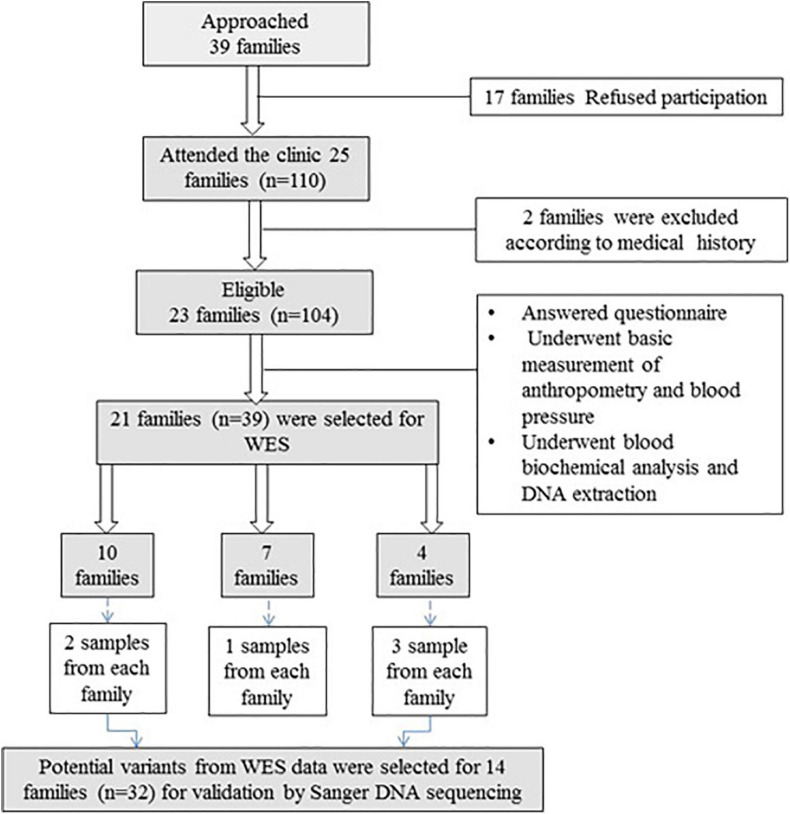
Flowchart of recruitment of families with vitD deficiency. Thirty-nine families were approached, and 21 families (*n* = 39) were included in WES. After variant filtration and prioritization, potential variants were validated in 14 families (*n* = 32) using Sanger DNA sequencing.

In total, 23 families (104 individual participants) with a history of vitD deficiency [serum 25(OH)D < 12 ng/ml] were recruited. Of these, 39 samples from 21 families were selected for WES (Figure 1). Exclusion criteria for inclusion in the WES analysis included history of chronic renal and liver disease, cancer, malabsorption syndrome, rheumatoid arthritis, intake of medications with possible effects on vitD (such as glucocorticoids and anticonvulsants), hyperthyroidism, hyperparathyroidism, diabetes, or any other endocrinal disorders.

### Study Procedure and Blood Analysis

All participants answered a questionnaire (filled by the researcher), which requested information including socio-demographic data, medical history, drug history, and lifestyle history. Each participant underwent basic anthropometric and blood pressure measurements. Multi-generation pedigree was carefully made for each family by interviewing the family and documenting the family history of vitD deficiency. Fasting blood samples of all members of the family and from 100 unrelated controls were collected. Total serum 25(OH)D and intact PTH were measured by chemiluminescence immunoassay (CLIA), using a LIAISON auto-analyzer (DiaSorin Inc., Stillwater, MN, United States); free 25 (OH)D was directly measured by immunoassay using ELISA kit (KAPF1991, Future Diagnostics Solutions B.V., Wijchen, Netherlands); and VDBP was measured by quantitative sandwich enzyme immunoassay using Quantikine^®^ ELISA (DVDBP0B, R&D Systems, Minneapolis, MN, United States). Serum albumin, Ca, PO_4_, magnesium (Mg), lipid profile, blood glucose, and renal and liver function were all measured by the colorimetric method using a VITROS 250 Clinical Chemistry auto-analyzer (Ortho-Clinical Diagnostics Inc., Rochester, NY, United States).

### Whole-Exome Sequencing

Genomic DNA was first extracted (DNA extraction kit 53104, Qiagen, Hilden, Germany), and the concentration and purity of the DNA filtrate were measured using a NanoDrop spectrophotometer (ND-1000 UV-VIS). WES with a 150-bp paired-end read length for 39 DNA samples was performed by next-generation sequencing (NGS) using the Illumina platform and Twist Human Core Exome library kit. Genomic DNA was extracted from all included blood samples, and a library was constructed by random fragmentation of DNA followed by 5′ and 3′ adapter ligation, or by “tagmentation” which coupled the fragmentation and ligation reactions in one step, increasing the proficiency of the library preparation procedure. Afterward, adapter-ligated fragments were PCR amplified and gel purified. The library was loaded into a flow cell so that fragments get captured on a lawn of surface-bound oligos complementary to the library adapters. Next, amplification of each fragment into different clonal clusters was done by bridge amplification. Once clusters were generated completely, templates were sequenced. Illumina SBS technology which uses a reversible terminator-based approach was utilized to identify single bases integrating into DNA template strands. This technology was used due to its lower rates of raw errors compared to other technologies, as natural competition in this technology due to the presence of all four reversible terminator-bound dNTPs during each sequencing cycle reduces incorporation bias. In addition, Illumine SBS produces very precise base-by-base sequencing that practically removes sequence-context-specific errors even within repetitive sequence regions and homopolymers. Sequencing data were transformed into raw data. Raw data or images were generated by the Illumina sequencer using integrated analysis software called Real Time Analysis which is a sequencing control software for system control and base calling. The base call binaries were converted into FASTQ by using Illumina package (bcl2fastq). Reads were produced without trimming away adaptors.

### Analysis of WES Data

Whole-exome sequencing data generated the raw reads in the form of FASTQ format. Insertion, deletion, and copy number variation were distinguished by utilizing SAMtools^1^. Data was aligned by using the BWA Aligner^2^, after the crude information FASTQ files were adjusted. The resulting VCF files contained over 120,000 variants per samples. The variants were clarified by using different parameters, such as quality, frequency, genomic position, protein effect, and association with vitD deficiency. SNPs or variants and short indel candidates were determined at nucleotide resolution. SNPs found were compared to 1000 genomes using the international genome^3^, SnpEff^4^, and gnomAD databases^5^. A bioinformatics tool (laser gene Genomic Suite v. 12, DNASTAR, Madison, WI, United States) was used to look for variants involved in vitD metabolism. Variant alleles were tagged according to dbSNP142 using ArrayStar v. 12 (Rockville, MD, United States). The obtained FASTQ sequences were aligned against the human reference genome using the Borrow--Wheel arrangement tool^6^ and reference genome hg19 for humans^7^. FASTQ raw data files were then transformed to BAM file format that were afterward annotated using Toolkit for Genome Analysis^8^. In this study, we targeted indels and SNPs situated in the exons and splicing junctions of the genes that caused protein-level changes, with exclusion of synonymous variants. Our selected variants were identified in around 45% of total reads.

### Variant Prioritization

For variant prioritization, the coding and splicing regions of genes involved in vitD metabolic pathways were analyzed and assessed using the available online database for these variants (see text footnote 5)^9^,^10^. Initially, variants positioned in introns, intergenic regions, and untranslated regions were excluded, as well as synonymous variants. To comprehend potential biological functions of the variants designated, the functional influence of the selected genomic variants and pathogenicity were evaluated using prediction algorithms (Mutation Taster, PolyPhen2, SIFT, PROVEAN, and Mutation Assessor) included in ANNOVAR^11^. Lastly, candidate genes were reviewed in PubMed publications and the Online Mendelian Inheritance in human’s database.

To analyze identified exonic variants related to vitD, we selected major genes involved in vitD metabolic pathways as follows: DHCR7, MC1R, GC, CYP2R1, CYP27B1, CYP24A1, VDR, RXRA, CUBN, LRP2, and CASR (Fischer, 2020).

After applying various filters, the total number of variants was reduced to 20–30 variants per sample. Finally, the variants involved in vitD metabolism were selected in the following target genes: GC, CUBN, LRP2, DHCR7, and CASR.

### Validation of WES Results by Sanger Sequencing

Validation of prioritized variants in candidate genes was conducted using Sanger sequencing. Designing primers were done initially using web-based Primer3 (v. 0.4.1) software. Primers used were as follows: GC gene: Forward: 5′-TGA GACAGGCAAGTATTTCTATT-3′ Reverse: 5′-GCCAAGTTA CAATAACACCAGGA-3′, CUBN gene: Forward 1: 5′-AGAG GATAAACAGTTAGGGGCT-3′ Reverse 1: 5′-GCAGAACCA GGACACAAAACT-3′ and Forward 2: 5′-CCGAACAGAGGA GGAGACC-3′ Reverse 2: 5′-ACGTTACATTTTATAGGCGT GA-3′ and for the LRP2 gene we used Forward 1: 5′-AAAAGG GGACAGGTACATACT-3′ Rreverse1: 5′-TCCTCCTCCACTAA TGCAACA-3′ Forward 2: 5′-TGTGCTCTGCTGTAGTGGAG-3′ Rreverse 2: 5′-TCTTGAGAAAACAGGCAAAGACA-3′ and for DHCR7 Forward: 5′-CTCATGTTCCTGCTATGCGTC-3′ Reverse: 5′-GACTGGCCCCTGAGAGAAAG-3′ and for CASR Forward: 5′-ACGGTCACCTTCTCACTGAG-3′ Reverse: 5′-GACAACTCTTCAGGGTCCTCC-3′. Next, PCR amplification and purification were performed for DNA samples and Sanger sequencing conducted using a genetic analyzer (3500 genetic analyzer, Applied Biosystems, Thermo Fisher Scientific, Waltham, MA, United States) and BigDye Terminator V3.1 Cycle Sequencing kit (cat#4337455, Applied Biosystems, Thermo Fisher Scientific, Waltham, MA, United States).

The validated results were compared with the results of control samples (*n* = 100). Controls were matched with index samples for age, skin tone, sunlight exposure, oral vitD intake, and BMI but notably were vitD sufficient.

## Results

### Results of WES Data

Various missense variants with moderate impact were determined in GC, CUBN, LRP2, DHCR7, and CASR genes (Table 1). The polymorphism rs9016 in GC was detected in 13 families (*n* = 30), rs1801726 in CASR was detected in 12 families (*n* = 28), while rs4667591 and rs2075252 in the LRP2 gene were observed in six families (*n* = 13) and three families (*n* = 6), respectively. In addition, rs1801222 and rs1801224 in CUBN and rs143587828 in DHCR7 were each detected in a different family (each family *n* = 2).

**TABLE 1:** Filtered WES results of vitD-related gene variants among vitD-deficient families.

**Gene name**	**Family ID (F#)**	**Chromosome**	**HGVS.c**	**HGVS.p**	**Effect**	**Putative Impact**	**SNP**
GC	F1, F2, F3, F4, F5, F6, F7, F8, F9, F10, F12, F13, F14	Chr4	1391A > G	His464Arg	Missense variant	Moderate	rs9016
CUBN	F5	Chr10	758T > C	Phe253Ser	Missense variant	Moderate	rs1801222
	F6		1165C > A	Pro389Thr	Missense variant	Moderate	rs1801224
LRP2	F1, F3, F9	Chr2	12280A > G	Lys4094Glu	Missense variant	Moderate	rs2075252
	F2, F5, F7, F10, F12, F13		12628A > C	Ile4210Leu	Missense variant	Moderate	rs4667591
DHCR7	F1	Chr11	376G > A	Val126Ile	Missense variant	Moderate	rs143587828
CASR	F1, F2, F3, F4, F5, F6, F8, F9, F10, F12, F13, F14	Chr3	3061G > C	Glu1021Gln	Missense variant	Moderate	rs1801726

*HGVS.c/p stands for Human Genome Variation Society nomenclature for DNA reference sequence or protein reference sequence, respectively. F1, F2, F3, F5, F7, F8, F9, F11, F12, and F13: *n* = 2 for each family group. F4, F6, F10, and F14: *n* = 3 for each family group.*

### Validation of WES Results

#### CUBN

**FIGURE 2:**
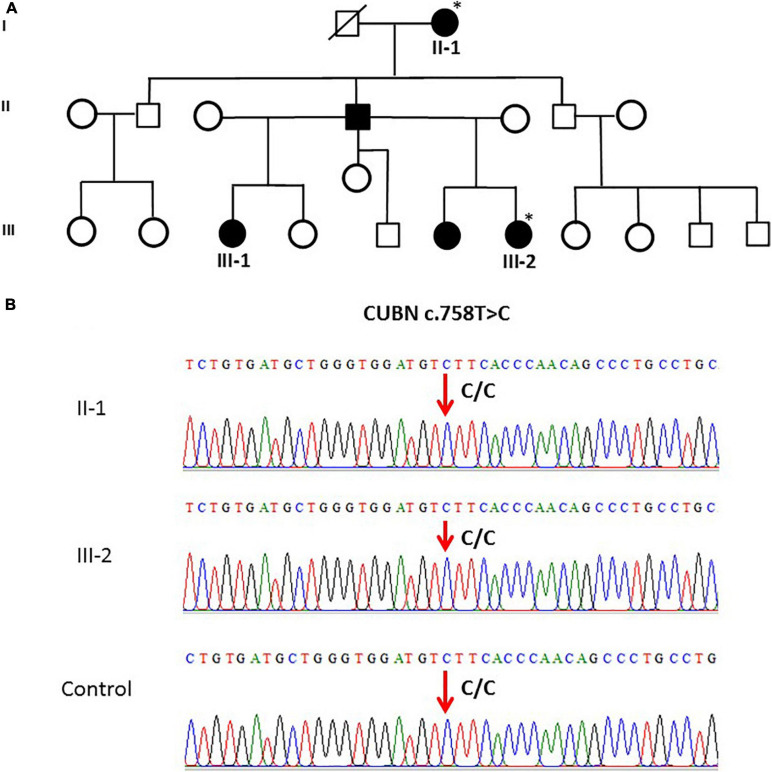
**(A)** A pedigree for family 5, showing vitD-deficient II-1 and III-2 subjects. **(B)** Sanger sequencing chromatogram revealed c.758T > C polymorphism (homozygous CC genotype) in CUBN gene in members of family 5 and control. ^∗^ Samples analysed.

The CUBN variant c.758T > C in family 5 (*n* = 2) was validated. Both family 5 samples and the controls were homozygous (CC genotype) as shown in Figures 2A,B.

#### LRP2

**FIGURE 3:**
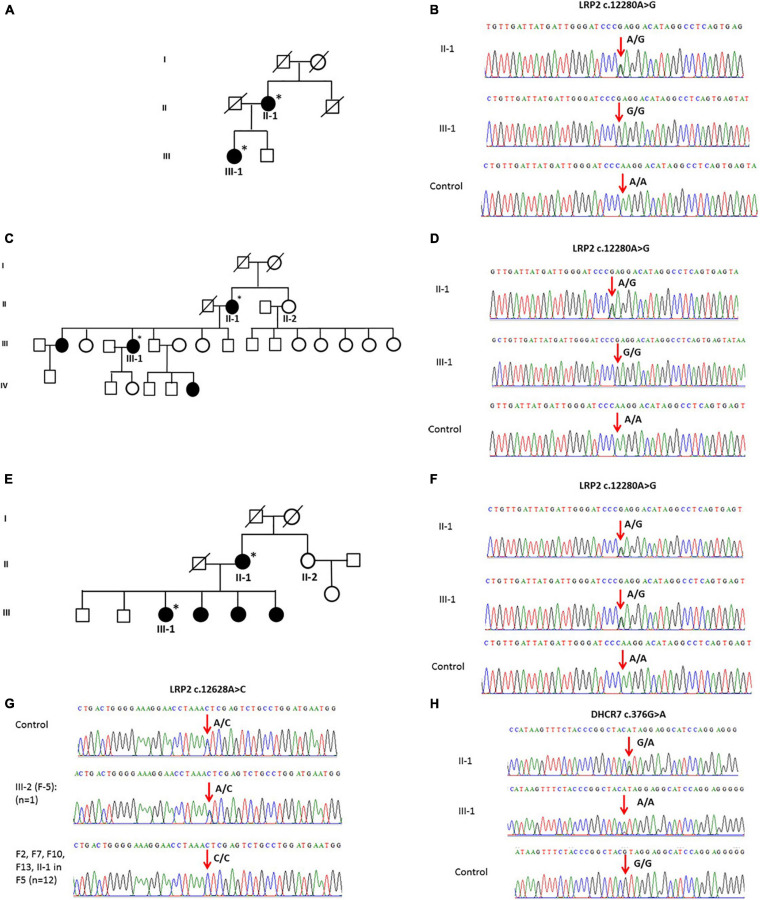
**(A)** A pedigree of family 1 representing II-1 and III-1 subjects with history of vitD deficiency. **(B)** Sanger sequencing chromatogram of family 1 showing the c.12280A > G variant in LRP2 with heterozygous AG genotype in subject II-1, homozygous GG genotype in III-1 subject, and homozygous AA genotype in control. **(C)** A multi-generation pedigree for family 9 showing the analyzed samples with vitD deficiency (the mother II-1 and daughter III-1). **(D)** Sanger sequencing chromatograms for c.12280 > C LRP2 variants in control (AA), mother (AG), and daughter (GG). **(E)** A multi-generation pedigree for family 3 showing the analyzed samples with vitD deficiency (the mother II-1 and daughter III-1). **(F)** Sanger sequencing chromatograms for c.12280 > C LRP2 variants in control (AA), mother (AG), and daughter (AG). **(G)** Examples of Sanger sequencing chromatograms representing the variant c.12628A > C in the LRP2 gene. Majority of samples (*n* = 12) were homozygous (CC), while control samples (*n* = 100) and a single sample in F5 were heterozygous. **(H)** Sanger sequencing chromatogram showing c.376G > A mutation in the DHCR7 gene as (G/A) in mother and (A/A) in daughter compared to control (G/G). ^∗^ Samples analysed.

The general and biochemical characteristics of families (F1, F3, and F9) that exhibited the c.12280A > G (rs2075252) variant in LRP2 are shown in Tables 2, 3. Validation of this SNP (rs2075252) showed that F1, F3, and F9 had this variant while the control did not. In family 1 and family 9, subject II-1 (the mother) had heterozygous AG genotype while subject III-1 (the daughter) had a homozygous GG genotype and the control samples had a homozygous AA genotype (Figures 3A–D). On the other hand, both subjects II-1 and III-1 in family 3 had the heterozygous AG genotype (Figures 3E,F).

**TABLE 2:** General characteristics of family 1, family 3, and family 9 female participants.

**Variables**	***Family 1***		***Family 3***		***Family 9***	
	***II-1***	***III-1***	***II-1***	***III-1***	***II-1***	***III-1***
Age (years)	70	54	59	35	65	40
Age at menopause years)	45	52	47	–	55	–
Years since menopause	25	2	12	–	10	–
Ethnicity	White (Arabic)	White (Arabic)	White (Arabic)	White (Arabic)		White (Arabic)
Weight (kg)	65	110	97	72	56	97
Height (cm)	140	149	161	167	149	158
BMI (kg/m^2^)	33.2	49.5	37.4	25.8	25.2	38.9
Waist circumference (cm)	90	119	100	128	90	110
Hip circumference (cm)	110	148	82	108	110	128
WHR	0.82	0.80	0.78	0.76	0.82	0.86
Hypertensive/Diabetic (according to medical records)	No	No	No	No	No	No
SBP (mmHg)	141	130	126	109	100	150
DBP (mmHg)	80	72	86	71	54	94
Marital status	Widow	Divorced	Widow	Married	Married	Married
Education	Illiterate	Secondary	University	University	Illiterate	Illiterate
Occupation	Housewife	Housewife	Self-employee	Government employee	Housewife	Housewife
Skin tone (Fitzpatrick)*	Type IV (olive and mid brown)	Type IV (olive and mid brown)	Type II (white and fair)	Type II (white and fair)	Type IV (olive and mid brown)	Type IV (olive and mid brown)
Sun exposure	<1 h/week	<1 h/week	<1 h/week	<1 h/week	1–2 h/week	1–2 h/week
Veiling type	Partially covered	Partially covered	Totally covered	Partially covered	Totally covered	Totally covered
Use of sunscreen	No	No	No	No	No	No
Dietary vitD intake (IU/day)	27	116	189	195	100	49
Use of vitD supplementation	Yes	Yes	Yes	No	No	No
Physical activity	No	No	No	Yes	No	Yes
Smoking	No	No	No	No	No	No

BMI, body mass index; WHR, waist–hip ratio; SBP, systolic blood pressure; and DBP, diastolic blood pressure. *Fitzpatrick scale Pike and Meyer, 2012. EAR is the estimated average requirement.

**TABLE 3:** Biochemical characteristics of family 1, family 3, and family 9 female participants.

**Variable**	***Family 1***		***Family 3***		***Family 9***	
	***II-1***	***III-1***	***II-1***	***III-1***	***II-1***	***III-1***
Serum total 25(OH)D (ng/ml)	7.8	10.1	16	13.4	17.2	14.3
Serum direct free 25(OH)D (pg/ml)	3.6	5.1	5.1	3.1	4.6	4.01
Percentage of free 25(OH)D out of total 25(OH)D (%)*	0.046	0.050	0.032	0.023	0.027	0.028
Serum VDBP (μg/ml)	350	402	192	332	554	664
Serum Albumin (g/L)	45	40	44	49	43	46
Serum Ca (mmol/L)	2.4	2.57	2.58	2.62	2.51	2.63
Serum PO_4_ (mmol/L)	1.34	1.19	1.34	1.36	1.42	1.46
Serum Mg (mmol/L)	0.8	0.9	0.9	0.9	0.8	0.8
Fasting blood glucose (mmol/L)	6.5	6.2	5.4	4.7	4.3	4.6
Serum AST (U/L)	25	17	20	21	22	21
Serum ALT (U/L)	33	25	20	17	21	18
Serum ALP (U/L)	105	116	76	60	66	62
Serum creatinine (μmol/L)	66	50	47	38	52	48
Serum total cholesterol (mmol/L)	6.2	5.7	2.9	4.1	4.9	5.3
Serum triglyceride (mmol/L)	2.30	1.58	0.59	1.12	1.2	1.08
Serum HDL-C (mmol/L)	1.2	1.3	1.2	1.2	1.7	2.2
Serum LDL-C (mmol/L)	3.8	3.72	1.39	2.37	2.1	2.58
Serum VLDL-C (mmol/L)	1.1	0.73	0.27	0.51	0.45	0.50

*Percentage of free 25(OH)D out of the total 25(OH)D was calculated by dividing free 25(OH)D levels in ng/ml over total 25(OH)D level in ng/ml, then multiplied by 100. 25(OH)D, 25-hydroxyvitamin D; VDBP, vitamin D-binding protein; Ca, calcium; PO4, phosphate; Mg, magnesium; AST, aspartate aminotransferase; ALT, alanine aminotransferase; ALP, alkaline phosphatase; HDL-C, high-lipoprotein cholesterol; LDL-C, low-density lipoprotein cholesterol; and VLDL-C, very low-density lipoprotein cholesterol.

The validation of the other polymorphism (c.12628A > C) in LRP2 that was observed with WES in F2, F5, F7, F10, F12, and F13 (*n* = 13) showed that this SNP existed in all the mentioned families and control samples (*n* = 100). All samples were homozygous CC except a single sample in F5 and four of the controls that were heterozygous AC (Figure 3G).

#### DHCR7

Whole-exome sequencing results showed variant c.376G > A in DHCR7 in Family 1 (F1). General and biochemical characteristics of F1 subjects were presented earlier in Tables 2, 3, and the pedigree of this family is shown in Figure 3A. Validation of the observed variant c.376G > A in DHCR7 in F1 revealed that subject II-1 (mother) has a GA genotype and II-1 has an AA genotype in comparison to the controls that had a GG genotype (Figure 3H). When this DHCR7 c.376G > A variant (rs143587828) was evaluated, it was found to be a mutation not a polymorphism.

#### GC

**FIGURE 4:**
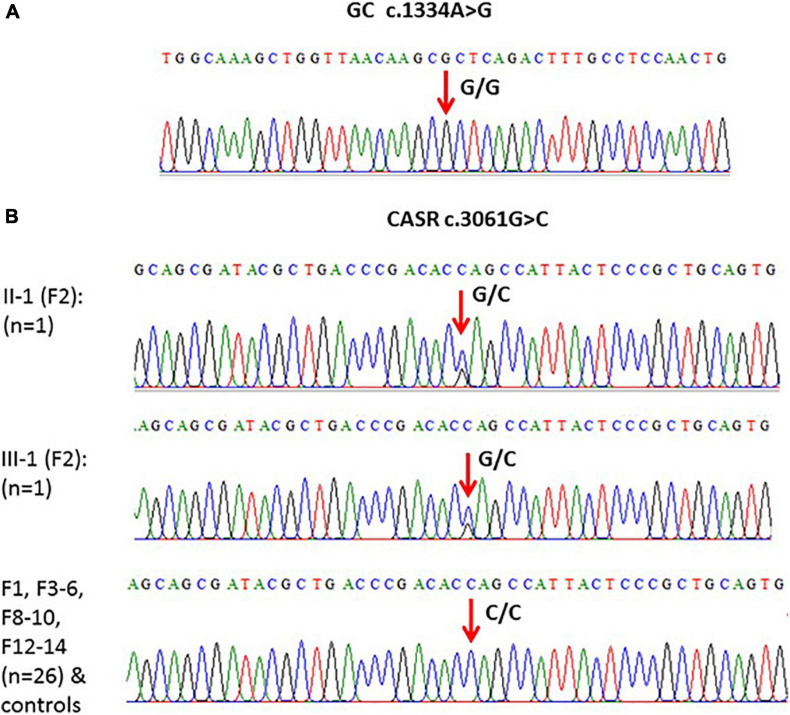
**(A)** An example of one of the chromatograms that showed c.1334A > G polymorphism (G/G) in the GC gene. **(B)** Models of elicited Sanger sequencing chromatograms that showed polymorphism (c.3061G > C) in the CASR gene. Homozygous CC was the major genotype, while heterozygous GC was found in F2 only.

When the WES results were validated by Sanger DNA sequencing for SNP c.1391A > G in GC in family samples (F1–F10 and F12–F14) (*n* = 30), the presence of c.1334A > G SNP as homozygous genotype (GG) was confirmed in these family samples as well as in the control healthy samples (Figure 4A).

#### CASR

Validation of the c.3061G > C variant in CASR in subjects from F1 to F6, F8 to F10, and F12 to 14 (*n* = 28) showed that this variant is present in the CC genotype in controls and in these families except F2 where the genotype was heterozygous (GC) (Figure 4B).

### Identified Polymorphisms and Mutations

In families with vitD deficiency, all observed variants were polymorphisms with the exception of the variant in DHR7 (rs143587828) which was a mutation. We found two single variants in LRP2 with one variant (rs2075252) observed in six individuals but not in control cases, while the other LRP2 variant (rs4667591) was detected in 13 subjects and in controls. A single variant in DHCR7 (rs143587828) and one in MC1R (rs1805005) were observed in two subjects from two different families but not in controls. Other variants in GC, CUBN, and CASR were found in index cases and controls. Polymorphisms in GC (rs9016) and CASR (rs1801726) were found in the majority of family cases (94% and 88%, respectively).

## Discussion

Several studies have linked vitD deficiency with numerous variants in genes involved in vitD metabolism (McGrath et al., 2010; Jolliffe et al., 2016). Our WES study in families having vitD deficiency revealed various variants in genes related to vitD; however, the majority of these variants including the ones in GC (rs9016), CUBN (rs1801222), CASR (rs1801726), and LRP2 (rs4667591) coexisted in both the vitD-deficient families and the non-affected control group (with GC and CASR SNPs having the highest frequency), suggesting no association between these SNPs and 25(OH)D levels. In agreement with our findings, a case–control study in Egyptians (*n* = 328) also found that CUBN (rs1801222) was not associated with total 25(OH)D levels Harding et al. (2006) and Elsabbagh et al. (2020) found no association between 25(OH)D and CASR (rs1801726). With regard to GC (rs9016) and LRP2 (rs4667591), no reports exist in the literature about their relationship with vitD. However, these two SNPs were reported in a family-based WES study specifically looking at SNPs in genes related to vitD metabolism in families with familial multiple sclerosis; however, no association was found with multiple sclerosis (Pytel et al., 2019).

In the present study, a mutation in DHCR7 (rs143587828) was identified in two affected subjects from one family (mother was heterozygous and daughter was homozygous for the minor allele) but not in any of the control subjects. As DHCR7 encodes for the production of the enzyme that is responsible for the conversion of 7-DHC (the precursor of vitD) to cholesterol (Berry and Hyppönen, 2011), it is suggested that this mutation in DHCR7 (rs143587828) might result in increased activity of DHCR7 leading to reduced conversion to vitD and thus vitD deficiency (Kohlmeier, 2012). Two large genome-wide association studies in subjects of European ancestry found that minor alleles of nine alternative SNPs in DHCR7/NADSYN1 were associated with vitD deficiency (Ahn et al., 2010; Wang et al., 2010). However, this may be the first report of the association of rs143587828 with 25(OH)D. This observed mutation in DHCR7 (rs143587828) now needs to be investigated in a large-scale population study, to explore further the association between this mutation and vitD status.

Cubilin and megalin, which are receptor proteins present in the proximal renal tubules encoded by CUBN and LRP2 genes, respectively, bind to the VDBP 25(OH)D complex and contribute to the process of endocytosis of the VDBP 25(OH)D complex so that 25(OH)D can be hydroxylated to 1,25(OH)_2_D, the active form of vitD (McGrath et al., 2010; Kaseda et al., 2011; Kohlmeier, 2015). Severe hypovitaminosis D was reported in LRP2 knockout mice, which suggests an important role for LRP2 (Nykjaer et al., 1999). In our study, we found an SNP (rs2075252) in LRP2 in six affected families (*n* = 13) but not in the controls. This strongly suggests that this SNP might be related to vitD deficiency and emphasizes the need for additional studies on the association between vitD status and SNPs in LRP2. To our knowledge, there is only one report in the literature and this opposes our finding, with polymorphism rs4667591 in LRP2 not found to be associated with total 25(OH)D (Elsabbagh et al., 2020).

Our study has revealed relevant and novel exonic missense variants in both DHCR7 and LRP2 in vitD-deficient families (not evident in control individuals); the association between these variants and vitD deficiency now needs to be addressed. Our results provide information on the variants related to vitD metabolism in families with vitD deficiency, thus helping researchers understand genetic factors underlying vitD deficiency in the Saudi population.

## Data Availability Statement

The datasets for this manuscript are not publicly available because family consents to share data publicly was not allowed. Requests to access the datasets should be directed to corresponding author (MN).

## Ethics Statement

Ethical approval of this study was obtained from the Research Ethics Committee in Unit of Biomedical Ethics, Center of Excellence in Genomic Medicine Research (CEGMR), King Abdulaziz University (KAU), (05-CEGMR-Bioeth-2018). Written informed consent to participate in this study was provided by the participants’ legal guardian/next of kin.

## Author Contributions

SA contributed to the study design and execution, data analysis, and manuscript drafting. MN contributed to the study design, data analysis, writing, editing, and review. EA and AC contributed to writing the review and supervision. MR contributed to the supervision and review of the manuscript. SL-N contributed to supervision. All the authors read and approved the final manuscript.

## Conflict of Interest

The authors declare that the research was conducted in the absence of any commercial or financial relationships that could be construed as a potential conflict of interest.

## Footnotes

1 http://samtools.Sourceforge.Net/

2 http://bio-bwa.Sourceforge.Net/

3 https://www.internationalgenome.org/

4 http://snpeff.sourceforge.net/SnpEff.html

5 https://gnomad.broadinstitute.org/

6 http://bio-bwa.sourceforge.net/bwa.shtml

7 http://hgdownload.cse.ucsc.edu/goldenPath

8 http://www.broadinstitute.org/gatk

9 http://www.1000genomes.org

10 http://www.ncbi.nlm.nih.gov/SNP/

11 http://www.openbioinformatics.org/annovar/
